# Fake kindness, caring and symbolic violence

**DOI:** 10.1177/09697330231209290

**Published:** 2023-10-25

**Authors:** Damien Contandriopoulos, Natalie Stake-Doucet, Joanna Schilling

**Affiliations:** 8205 University of Victoria; 5622 Université de Montréal; 8205 University of Victoria

**Keywords:** Caring, fake kindness, nursing, sociological theory, symbolic violence

## Abstract

The article starts by offering a definition of fake kindness focused on the dissociation between the behavioural components of kindness and the intent to sincerely pay some heed to the needs of others. Using the sociological theory of Pierre Bourdieu, this definition is then used to articulate how fake kindness can be conceptualized as a specific form of symbolic violence. Such a view allows explanations as to how and why the prevalence and effectiveness of fake kindness vary according to microsociological norms and values. The generic definition and conceptualization of fake kindness as a form of symbolic violence are then used to discuss how nursing’s enthrallment with the concept of caring and its operationalization as a moral compass likely fosters the growth of fake kindness within the profession. In this view, the institutional enforcement of propriety and well-behaved professionalism is more likely to lead to toxic environments than to healthy workplaces. We hope that being able to understand how professional norms and institutional rules are sometimes turned into social tools to enforce obedience and existing hierarchies can empower victims of those phenomena to resist them more effectively. It might also contribute to increasing the awareness of well-meaning nurses or people in position of authority who have been socialized in environments where fake kindness is normalized.

## Introduction

In 2010, Phillips and Taylor published a short book aptly titled ‘On Kindness’ whose main thesis is that contemporary Western societies evolved into framing kindness as a moral weakness: ‘*Most people*, *as they grow up now*, *secretly believe that kindness is a virtue of losers*’.^1^ This view suggests that we live in societies where people would likely try to hide public displays of kindness.

However, our own understanding differs. What we see around us is a world where displays of kind-looking behaviours are ubiquitous but largely disconnected from actual kindness. In this paper, we argue that there is a twin social trend of, on the one hand, overemphasizing the importance of displays of kind-looking behaviours while, on the other hand, discounting the value of actually paying heed to the needs of others.

We interpret this disconnection as a weaponizing of displays of kind-looking behaviours in symbolic struggles. This weaponization takes multiple forms – such as aggressive tone policing – that have in common to impose a narrowing down of legitimate forms of expression and an appropriation of legitimacy for purposes that have nothing kind about them. In other words, fake kindness is a form of symbolic violence^2,3^ used by dominant agents to strengthen their control on others.

We also believe that this weaponizing of fake kindness in social relations isn’t evenly distributed throughout the social space. For reasons we discuss here, fake kindness is more prevalent in some fields and contexts. Among those contexts, healthcare and education professions and institutions are especially propitious. We take the special case of nursing to explore how seemingly innocuous professional norms and values can turn out to be a nurturing ground for toxic behaviours.

Overall, we hope to provide a more sophisticated understanding of the role the strategic use of public displays of kindness play in social struggles. We also believe that our conceptual clarification and analysis of fake kindness can eventually help victims of that symbolic violence to self-defend.

## Defining fake kindness

The extent and prevalence of an innate human affinity for kindness has been a religious and philosophical preoccupation for a very long time.^1^ But despite its importance in day-to-day language and sense-making, the concept of kindness has remained somewhat peripheral in academic debates.^4,5^ At the social and scientific levels, the focus has instead generally been on somewhat related concepts such as empathy and altruism. At the individual level, some validated scales and instruments exist^5,6^ but again, the concept hasn’t been attracting a huge level of attention. Without any ambition to summarize existing knowledge on the concept, for the purpose of our argument, we propose that kindness can be split into two components. One is behavioural and the other teleological.

We define behavioural kindness as including all that one can observe from the outside: Ways of looking at others and listening to them, the tone and voice used, the language and the overall body posture. From an ethological point of view, behavioural kindness could be defined as the sum of all human readable signs of non-aggression. In addition, as outside signs of non-aggression will vary throughout time, places and culture, there certainly are also cultural and social components that go beyond the ethological and relate to social structuring.^2^ But, overall, behavioural kindness has to do with communicating intents.

At least in theory, one could perform acts of kindness while displaying no external signs of doing so, or even while displaying external signs of aggression. However, socially, such behaviour would be an exception rather than the rule. In her analytical typology of the related concept of empathy, Read^7^ distinguishes three main dimensions. The first is about sharing another person’s affective state and feelings, the second about consciously understanding those sentiments and the third is about responding appropriately. In the same way, the effective communication of intents is relevant to kindness at two levels. First, understanding another person’s kind dispositions towards oneself could foster social connections which have an intrinsic benefit. Second, kindness often – though not always – takes place through social interactions where all agents are involved in outcomes. To take a trivial example, even if very thirsty, one might refuse a glass of water provided by someone one believes to be a foe. Understanding other’s intentions and responding appropriately therefore likely matters to the social effectiveness of kindness too. Nevertheless, a central point we want to make regarding this first component of kindness is that it does not go beyond appearances and social communication.

Teleological kindness, on the other hand, includes all the values and intentions that support and guide kind actions. It concerns itself with the why and what of kindness. Teleological kindness is a central human virtue and has been treated and discussed as such for millenniums. Aristotle’s Rhetoric is often quoted for this definition of kindness mainly framed around selflessness: ‘*Kindness – under the influence of which a man is said to* “*be kind*” *– may be defined as helpfulness towards someone in need*, *not in return for anything*, *nor for the advantage of the helper himself*, *but for that of the person helped*’.^8^ In the same text, Aristotle then proceeds to specify that selflessness is central to the notion of kindness as helping others in the context of a reciprocal exchange or with selfish motivations does not qualify. Similar ideas can be found in many philosophical and religious perspectives. For example, Maimonides emphasizes that giving to those in need without knowing to whom one gives and when the recipient doesn’t know who gave belongs to the higher levels of kindness. In the same way, the centrality of selflessness and of a generic trust in humankind remains central in contemporary definitions. ‘*Real kindness is an exchange with essentially unpredictable consequences*. *It is a risk precisely because it mingles our needs and desires with the needs and desires of others*, *in a way that so-called self-interest never can*’.^1^ In our view, how selfless one needs to be in order to be kind is an open question. One could argue that the neutral point might be non-maleficence and that anything on the helpful side of it qualifies as kindness. This question is especially important when it comes to professional expectations. We believe that once kindness is defined as selflessness, it probably can’t be part of one’s job description. But, for many professionals, non-malevolence (e.g. expressed as *primum non nocere*) is a core expectation.

The central point we want to make here is about the distinction between the behavioural and teleological components of kindness. For reasons we will explore, some people adopt the behavioural components of kindness without having any teleological interest in being kind. *Our definition of* ‘*fake kindness*’ *relates to this instrumental dissociation of the behavioural and teleological components of kindness*. As we will argue in more detail below, when dissociated in such a way, displays of behavioural kindness should be understood as a social tool aimed at modifying the behaviour of others. We also want to emphasize that there isn’t anything kind about fake kindness.

Interestingly, when Paley^9^ discusses compassion in the context of nursing, he makes a distinction between two components of the concept. He suggests that compassion is both a behaviour – defined here as actual actions that are compassionate – and a motivation – the intent to be compassionate – and that one component does not imply the other. Paley’s components of compassion are quite different from ours when defining kindness, but they connect to the same principle that behaviours and intents should not be presumed to be easily inferred one from the other.

The level of dissociation is also meaningful in itself. Some displays of fake kindness will push the behavioural signs to lengths that are almost caricatural. The voice will be oddly unctuous, the tone will be kept to extreme non-assertiveness and the smile will be rigidly kept on. Overdone behavioural signs of kindness are, in our experience, correlated with the extent to which the content or implications of the statements are actually harmful to others.

In popular culture, one well-known example of a caricatural dissociation between the behavioural and teleological components of kindness would be the fictional character of Dolores Umbridge from the Harry Potter novels. Under Umbridge’s lavish girlishness, unctuous manners and smiles, lies a mildly sadistic person and one of the book’s most-despised villains. Another fictional character that embodies the concept of fake kindness with blood-curling realism would be Nurse Ratched from One Flew Over the Cuckoo’s Nest. Ratched is a stern but soft-spoken character always acting like she is concerned by the wellbeing of the patients under her care and acting in their best interests. However, as the movie unfolds, it becomes obvious that this pretence and her motherly concerns are artifices to impose her ruthless and tyrannical rule on everyone. In recent public events, many commenters have also used the term fake kindness to describe the approach of Dr. Bonnie Henry, the provincial health officer of British Columbia during the COVID-19 pandemic. Dr. Henry’s trademark ‘be kind’ motto, smiles and soft voice were instrumental to her success, notwithstanding the fact many, like the provincial Human Rights Commissioner, expressed deep concerns about the ultimate impact of her decisions.^10^ In the same way, some have criticized how the French president, Emmanuel Macron, instrumentalized the term kindness (*bienveillance* in French) to impose hotly contested policies.^11^

It might also be worth emphasizing that fake kindness isn’t really an effort at deception. In most situations, the agents involved will rapidly decode that there is a silent whip cracking behind the unctuous voice. In that sense, fake kindness is different from hypocrisy – or what Beard^12^ describes as performative kindness – as everyone involved is likely aware that no sincere efforts at selflessness exist.

## Fake kindness as symbolic power

A core characteristic of fake kindness is that it is mostly used by dominant agents in social relations that are already asymmetrical. As a strategy, fake kindness only really makes sense for agents who can rely on underlying social or organizational structures to grant power to their words.^13,14^ Imagine a supervisor meeting an employee to inform them they will be transferred to a less desirable position, or a public health officer announcing that efforts at protecting vulnerable populations will be abandoned. In those examples, the discursive situation makes the words spoken highly performative in themselves. In such contexts, the capacity to convince others may not be that important and, hence, the usual rhetorical tricks might not be favoured by the speaker. On the other hand, as we will discuss, the speaker’s reliance on external displays of behavioural kindness can prove an effective tool to deprive hearers from otherwise legitimate responses of resistance.

Fake kindness is used by dominant agents to force their counterparts to limit themselves to similar behavioural boundaries: soft-speaking, well mannered, ‘professional’ and courteous. But, precisely because those exchanges are heavily asymmetric, the social effectiveness of civility won’t be fairly shared. The supervisor firing an employee in a calm and courteous way can impose their will. But the employee can’t reply in a calm and courteous voice to state that they will keep their job and expect it to work. In such a hypothetical exchange, the supervisor is using fake kindness as a social trap to impose obedience on the employee. Behavioural deviations (shouting, crying, swearing, etc.) would then be construed as indications that the employee is conflictual, unprofessional, or dysfunctional and further prove the appropriateness of the termination. Fake kindness heavily relies on tone-policing^15,16^ and those using it can generally also rely on institutional rules and structures that will enforce tone-policing in disciplinary proceedings.

As such, fake kindness is essentially a way for dominant agents to force on their counterparts a passive acceptance of their subordination by limiting the behavioural repertoire available to them.^17^ In the same way, displays of behavioural kindness pushed to quasi-caricatural lengths while the absence of any teleological good intentions is also made abundantly clear – should be understood as social signalling. They are ethological warnings from the dominant to the subordinate that any behavioural deviation from passive obedience will be harshly sanctioned.

French sociologist Bourdieu^18^ coined the term symbolic violence to describe how social norms and institutional rules are intertwined in a way that implicitly, but forcefully, constrain individual behaviours within asymmetric social hierarchies. His theory^2,3,18^ conceives human agents as being socialized to – more or less consciously – integrate such social hierarchies and structure their behaviours accordingly. As such, symbolic violence is both a tool of social discipline and a manifestation of internalized hierarchies.

It is also important to keep in mind that social spaces – or maybe more accurately fields in the Bourdieusian sense^14^ – will not all value behavioural kindness to the same extent. The social expectations regarding agents’ behaviour in education or healthcare settings will be different than those for people involved in business transactions or political activities. Primary school teachers or nurses,^9^ for example, are heavily socialized around the desirability of external displays of behavioural kindness.

As some fields value and emphasize the desirability of behavioural kindness to different extents, the symbolic value associated with it will vary accordingly. In Bourdieusian terms, the symbolic capital one can gain from displaying the attributes of behavioural kindness will be field-dependent. A nurse is likely to be able to gain a significant level of symbolic capital by displaying the attributes of behavioural kindness because the institution they work in, and their colleagues will construe those displays as examples of professionalism. The fictional character of Nurse Ratched, for example, clearly instrumentalizes behavioural kindness as a source of institutional support and power. On the other hand, a foreman working in an industrial setting would likely gain much less symbolic capital using the exact same behaviours.

The elephant in the room that we skirted around so far is the gendered component of fake kindness. Whether kindness itself is gendered is a question we will leave for others to discuss. But we believe that many characteristic displays of behavioural kindness are clearly gendered as feminine. We recognize this has resulted in centuries of gender-based criticism and that at present, certain professions and people who identify as female in high-profile roles are particularly vulnerable to gender-based attacks. Further, we reject the idea that characteristics of behavioural kindness should be viewed as a weakness. However, acting in bad faith is not bound to an agent’s gender. In a society striving for equality, one’s gender or profession should not remove the possibility of a critical inquiry into their behaviour and motivations.

At the individual level, there are well-documented gendered social expectations about the ‘proper’ balance between assertiveness and meekness for people who identify as female. On the one hand, these expectations can result in a significant barrier to achieving gender equality. But on the other, this might create the opportunity for some to strategically use fake kindness to advance their own interests. At the collective level, those gendered norms will, in turn, increase the effectiveness and prevalence of fake kindness in traditionally feminine fields and professions. As with all socially structured behaviours, the interdependence of individual actions and social norms are complex and probabilistic.^2,3,14^ Therefore, describing behavioural kindness as a gendered expectation says little about the respective way a person who identifies as male and professionally socialized in a feminine field such as nursing or childcare or a person who identifies as female and professionally socialized in a masculine field such as surgery or carpentry, might act.

We also want to stress that affirming, as we do, that fake kindness is a gendered expression of symbolic violence does not imply in any way that symbolic violence in itself is something feminine. The opposite is much more likely. Symbolic violence is a concept dealing with the ways existing power imbalances and rules of dominance are expressed, enforced and maintained. In patriarchal societies like ours, people who identify as female are overall much likelier to be on the receiving end of symbolic violence.^19^ The point we are making here is limited to stating that one specific expression of symbolic violence – among others – is intertwined in gendered social norms.

## Nursing’s problem with fake kindness

As we suggested in the previous section, there are field-level sociological factors which likely affect both the effectiveness and prevalence of fake kindness and we believe a lot of health and social professions are particularly propitious to it. Our focus here, however, will mostly be on nursing as it is a profession with a somewhat perplexing relation with the concept of caring^20^ as well as pervasive issues with bullying and horizontal violence.^21–23^ Those two characteristics of nursing have been extensively discussed but are generally conceived as unrelated. By contrast – and in line with similar arguments proposed by Walker^24^ as ‘*the tyranny of niceness*’ and by Burton^25^ as ‘*the caring tax*’ – we think that understanding fake kindness as a form of symbolic violence might provide insights into how the discipline’s obsession with the concept of caring and its extensive problems with bullying actually connect.

Nursing is a profoundly gendered and hierarchical profession. In Anglo-Saxon countries, the roots of present-day nursing are linked to the sexist and racist values of Victorian Britain. Those roots also connect to the historic – and gendered – subordination of nursing to medicine in ‘modern’ hospitals.^26,27^ Despite the discipline’s rebranding efforts, today’s nursing still bears the marks of those influences.^27,28^ In academia, propriety and weaponized civility often continue to take precedence over independent thought and critique: ‘*The fiefdoms of the elite are critique-free zones*, *virtually devoid of any spirit of inquiry*, *ethos of debate or culture of scholarship*’.^29^ In that context, our understanding is that professional expectations are often used as tools to sustain traditional hierarchy and disciplinary power. The same can be said of the discipline’s quasi deification of Florence Nightingale. Nightingale believed a nurse must develop the proper moral standing to be considered good, which would imbue her with the power to police and discipline. Women from the middle and upper classes were hired as a disciplinary tool for their supposed superior moral influence that was thought to mould new nurses into the characteristics of the ideal nurse: quiet, obedient, and nurturing.^30^

The concept of caring emerged nearly a century after Nightingale’s nursing reformation as a way to establish nursing as a unique discipline with its own body of knowledge.^31^ Professional caring is both an action, to provide care, and a trait, to be caring. However, the discipline’s definition of caring has, over decades of endless debates, evolved into a fully normative concept that describes everything nursing-related that is good and desirable. The corollary of this definition being that the notion is also tautological: criticizing the concept of caring is uncaring and therefore unworthy of a good nurse.^32^ The overlaps in the concepts of caring and kindness are also hard to miss. For example, caring is generally framed around the motivation to act such as selflessness and sacrifice.^32,33^ In the same way, caring often prescribes specific behaviours or even scripts that nurses must adhere to be considered ‘good’ nurses and that overlap to a large extent with what we described as behavioural kindness.

But notwithstanding its reincarnation as a moral virtue, to this day, caring remains foundational to nursing as an academic discipline. Some of nursing’s most revered and cited scholars, such as Jean Watson, are theorists of caring. There are entire scientific journals dedicated to the topic, like the *International Journal for Human Caring*. And, at least in North America, caring plays a central role in curriculum content and structure. We know of no other profession that puts such a strong emphasis on moral values and proper behaviour.^9,33^ And while we agree that the image and place of nursing have been deeply shaped by external social forces, the discipline itself has continued to perpetuate the virtue script: nurses are good women who are honest, kind, caring, compassionate and self-sacrificial. For example, recruitment campaigns have attempted to attract women into the profession by promoting traditional stereotypes of nurses as ‘women born to be good’.^34^ In the same way, in 2021, the Canadian Nursing Association’s national nursing week theme was ‘*We answer the call*’^35^ reinforcing the belief that nursing is first and foremost a vocation.

According to the framework we developed in the previous sections, the emphasis nursing puts on caring as a behavioural and moral compass has implications in the way it shapes the sociological field in which nurses work. Among other things, this emphasis will increase the symbolic value associated with behavioural kindness to a level that is somewhat unique. In other words, nowhere will it pay more to behave in ways that align with behavioural kindness than it does in nursing. This isn’t a bad thing in itself. For example, displays of behavioural kindness at the bedside might actually benefit patients. At a first level it might bring some patients a sense of relief through emotional empathy.^7^ But even when used more instrumentally as a tool aimed at modifying patient behaviour and fostering compliance, it is possible to imagine scenarios in which that compliance would be aligned with the patient’s wellbeing. However, such benevolent scenarios imply that there is a convergence rather than a dissociation between behavioural and teleological kindness and this won’t always be the case. But the point we want to make is that the symbolic value associated with behavioural kindness in nursing frames the profession as a specific social field in which both the prevalence and effectiveness of fake kindness will be extremely high.

This being said, we see no reason to believe there is more symbolic violence exerted in nursing than in any other social field. However, we do think that nursing’s professional and disciplinary enthusiasm for caring will shape the form that symbolic violence, and more generally power struggles, will take. Instead of transparent displays of domination and direct confrontation, power struggle will be played in the register of fake kindness. Burton^25^ aptly summarizes this when she writes: ‘*This sensibility is sanctified in our culture in the notion that a good woman does not contradict and a nice woman does what she is told*’. Something that will allow nurses in hierarchical positions to assert their dominance without having to fear the pushback that only a ‘nasty’, ‘bad’ and ‘uncaring’ nurse would offer. In that sense, the discipline’s requirements for propriety, professionalism and civility – generally all bundled into, and implied by, the caring ethos – are the mainstay upon which the effectiveness of fake kindness rests.

## Conclusion

Our main point in this article is that fake kindness is a specific form of symbolic violence through which the dominants assert their power in asymmetric social relations. The effectiveness of fake kindness generally doesn’t rest on deception – fooling victims into believing that they are facing a well-meaning interlocutor – but on limiting the social repertoire victims can use. Those on the receiving end of fake kindness might not gain much from being able to properly name their experience. However, from time-to-time, the labelling of social and psychological tricks used to manipulate others – such as gaslighting – does help victims to gain a better understanding of what is happening and to explain it to others. As such, our hope is that this article might help some victims of fake kindness to respond in the best possible way to the symbolic violence they are exposed to.

The nature of fake kindness also makes it a sneaky expression of symbolic power whose effectiveness rests for a large part on institutional complicity to support and sustain it. Professional organizations and regulators, academic settings and care delivery institutions that incorporate and enforce strong civility and propriety requirements will tend to boost both the prevalence and effectiveness of fake kindness. This conclusion has direct implications for practice. Fostering respectful environments require more than mechanistic rules about what behaviours are allowed or sanctioned. If their goal is really to foster a type of respect that goes beyond the obedience of the oppressed, those in positions of power should be very careful about the net impact of their expectations and behaviours regarding civility and propriety.

In our view, the conceptualization of fake kindness that we developed is especially relevant for nursing. The profession’s enthrallment for caring as a behavioural and moral compass constitutes it as a social field especially propitious to fake kindness. While proponents of the caring mantra likely believe they are supporting a healthy and supportive professional and institutional culture, we do not share this optimistic view. The structuring of social expectations and rules that the caring culture fosters might have more to do with maintaining existing hierarchies and enforcing obedience than with anything else. We believe that professions which create the underlying conditions for fake kindness to be effective and prevalent will also foster a context propitious to bullying. As Walker wrote ‘*The idea that a* ‘*good nurse is a nice nurse*’ […] *is no longer viable in the 21st century*’.^24^ Our own opinion is that, on its own the concept of caring outlived its usefulness. We do not expect everyone to agree but we hope that our critical analysis of the potential connections between caring, fake kindness and bullying could contribute to productive debates within the profession.

## References

[1] Phillips A Taylor B . On kindness. London, UK: Picador, 2010.

[2] Bourdieu P . Outline of a theory of practice. Cambridge, UK: Cambridge University Press, 1977.

[3] Bourdieu P . Raisons pratiques sur la théorie de l’action. Paris, France: Éditions du Seuil, 1994.

[4] Brownlie J Anderson S . Thinking sociologically about kindness: puncturing the blasé in the ordinary city. Sociology 2017; 51(6): 1222–1238.

[5] Comunian AL . The kindness scale. Psychol Rep 2016; 83(3_suppl): 1351–1361.

[6] John OP Srivastava S . The big-five trait taxonomy: history, measurement, and theoretical perspectives. In: Pervin LA John OP (eds) Handbook of personality: theory and research. New York, NY: Guilford Press, 1999, pp. 102–138.

[7] Read H . A typology of empathy and its many moral forms. Philos Compass. 2019; 14(10): 1–12.

[8] Aristotle . rhetoric: an ancient Greek treatise on the art of persuasion (Translated by W. Rhys Roberts). 4th century. BCE, 2015, p. 134.

[9] Paley J . Cognition and the compassion deficit: the social psychology of helping behaviour in nursing. Nurs Philos 2014; 15(4): 274–287. 24447716 10.1111/nup.12047

[10] Ruttle J . End of health-care mask mandates puts vulnerable at risk: B.C. human rights commissioner. Vancouver, BC: Vancouver Sun, 2023.

[11] Viktorovitch C . L’appel à la bienveillance, une forme de violence? Paris, France: France Info, 2023. Available from: https://www.francetvinfo.fr/replay-radio/entre-les-lignes/chronique-l-appel-a-la-bienveillance-une-forme-de-violence_6027371.html

[12] Beard M . The difference between genuine kindness and fake kindness. 2019, 18 April, 2023. Available from: https://www.abc.net.au/everyday/the-difference-between-genuine-and-fake-kindness/11737836

[13] Bourdieu P . Language and symbolic power. Cambridge, UK: Polity Press, 1991.

[14] Bourdieu P . Espace social et genèse des «classes». Actes Rech Sci Soc. 1984; (52/53): 3–14.11634459

[15] Bailey A . On anger, silence, and epistemic injustice. London, UK: Royal Institute of Philosophy Supplements, 2018, 84: 93–115.

[16] Biddle C Hufnagel E . Navigating the “danger zone”: tone policing and the bounding of civility in the practice of student voice. Am J Educ 2019; 125(4): 487–520.

[17] Lorde A. The uses of anger: women responding to racism. 1981. In: Paper presented at the National Women’s Studies Association Conference, Storrs, Connecticut. Available from: https://www.blackpast.org/african-american-history/speeches-african-american-history/1981-audre-lorde-uses-anger-women-responding-racism/#sthash.IaK5NXKi.dpuf (accessed May 9, 2023).

[18] Bourdieu P . Distinction: a social critique of the judgement of taste. Cambridge, MA: Harvard University Press, 1984.

[19] Bourdieu P . La domination masculine. Collection Liber. Paris, France: Seuil, 1998.

[20] Paley J . Caring as a slave morality: nietzschean themes in nursing ethics. J Adv Nurs 2002; 40(1): 25–35. 12230525 10.1046/j.1365-2648.2002.02337.x

[21] Blackstock S Salami B Cummings GG . Organisational antecedents, policy and horizontal violence among nurses: an integrative review. J Nurs Manag 2018; 26(8): 972–991. 30171643 10.1111/jonm.12623

[22] Kiprillis N Gray R Robinson E , et al. Prevalence of horizontal violence of nurses in their first year of practice: a systematic review. Collegian 2022; 29(2): 236–244.

[23] Vessey JA Demarco R DiFazio R . Bullying, harassment, and horizontal violence in the nursing workforce: the state of the science. Annu Rev Nurs Res 2010; 28: 133–157. 21639026 10.1891/0739-6686.28.133

[24] Walker K . Whither nursing? Reflections on fate and futurity. Contemp Nurse 2003: 16(1–2): 3–8. 14994891

[25] Burton CW . Paying the caring tax: the detrimental influences of gender expectations on the development of nursing education and science. ANS Adv Nurs Sci 2020; 43(3): 266–277. 32732607 10.1097/ANS.0000000000000319

[26] Foucault M . Discipline and punish: the birth of the prison. New York, NY: Vintage Books, 1995.

[27] Rosenberg CE . The care of strangers: the rise of America’s hospital system. New York, NY: Basic Books Inc, 1987.

[28] Reverby SM . Ordered to care: the dilemma of American nursing, 1850–1945. Cambridge, UK: Cambridge University Press, 1987.

[29] Thompson DR Darbyshire P . Is academic nursing being sabotaged by its own killer elite? J Adv Nurs 2013; 69(1): 1–3. 23252796 10.1111/j.1365-2648.2012.06108.x

[30] Hawkins S . Nursing and women’s labour in the nineteenth century: the quest for independence. New York, NY: Taylor & Francis Group, 2010.

[31] Risjord M . Nursing knowledge: science, practice, and philosophy. Oxford, UK: John Wiley, 2010.

[32] Stake-Doucet N . Newspeak to nursespeak: leadership is compliance. In: Contandriopoulos D , et al. (eds) [pɔ ka se] Un recueil des post publiés sur le site de la chaire politiques connaissances et santé 2015–2019. Victoria: Les Éditions de l’Autruche, 2021, pp. 144.

[33] Dahlke S Stahlke Wall S . Does the emphasis on caring within nursing contribute to nurses’ silence about practice issues? Nurs Philos 2017; 18(3): e12150. 10.1111/nup.12150 27699966

[34] Gordon S Nelson S . An end to angels. Am J Nurs 2005; 105(5): 62–69. 10.1097/00000446-200505000-00031 15867536

[35] Canadian Nurses Association . We answer the call (facebook ad). 2021, Available from: https://m.facebook.com/CNA.AIIC/videos/weanswerthecall/860690731380609/

